# Maternal Mortality from Induced Abortion in Malawi: What Does the Latest Evidence Suggest?

**DOI:** 10.3390/ijerph181910506

**Published:** 2021-10-07

**Authors:** Calum Miller

**Affiliations:** St. Benet’s Hall, University of Oxford, Oxford OX1 3LN, UK; c.miller@oxon.org

**Keywords:** abortion, Malawi, spontaneous abortion, maternal deaths, maternal mortality

## Abstract

It is commonly claimed that thousands of women die every year from unsafe abortion in Malawi. This commentary critically assesses those claims, demonstrating that these estimates are not supported by the evidence. On the contrary, the latest evidence—itself from 15 to 20 years ago—suggests that 6–7% of maternal deaths in Malawi are attributable to induced and spontaneous abortion combined, totalling approximately 70–150 deaths per year. I then offer some evidence suggesting that a substantial proportion of these are attributable to spontaneous abortion. To reduce maternal mortality by large margins, emergency obstetric care should be prioritised, which will also save women from complications of induced and spontaneous abortion.

## 1. Introduction

Malawi is a democratic republic of 19 million people in southeastern Africa. It is a majority Christian country with a significant minority of Muslims. The population is largely rural, and it is generally considered a low-income country, with a high maternal mortality ratio. Because the maternal mortality ratio is so high, it has been wondered whether legalising abortion might provide a partial solution. Attempts to legalise abortion recently failed, as the Malawian parliament unanimously rejected a proposed bill in early 2021, and a later bill was withdrawn by the sponsor [1,2].

It is commonly claimed that extremely large numbers of women die from illegal induced abortion in Malawi each year, and that these illegal, unsafe abortions would be converted to safe abortions upon legalisation, hence lowering the maternal mortality ratio. Given the central role that claims about women dying from unsafe abortion play in the abortion debate in Malawi and around the world, it is critically important that both sides have a clear understanding of the existing research on this question rather than relying on outdated or misleading figures, or exaggerated claims in the media. These figures are often misleading because deaths from ‘abortion’ standardly include deaths from induced and spontaneous abortion combined, without distinguishing the two. They also typically include legal and illegal abortion without distinction. Interpreting all of these deaths as being attributable to illegal abortion alone will lead to an overestimate—perhaps a large overestimate—of deaths attributable to illegal abortion.

It is important that an accurate assessment of maternal mortality from unsafe abortion is made, for a number of reasons. First, it will help prioritise resource allocation in order to reduce maternal mortality as much as possible. Second, it will inform policy debates regarding the legal status of abortion: if far fewer women die from unsafe abortion than typically suggested, and if the evidence that legalising abortion would prevent these deaths is sparse, then the argument for legalising abortion on the basis of maternal mortality is correspondingly weakened. The same arguments on both sides apply across much of the developing world.

While it is uncontroversial that some women die from illegal induced abortion each year, this article demonstrates that the latest evidence shows the number to be far fewer than commonly claimed. I first discuss the most recent estimates for total maternal deaths, then the most recent estimates for maternal deaths attributable to induced abortion and spontaneous abortion combined, before offering some comments on the distinction and identification of illegal induced abortions specifically.

## 2. Total Maternal Deaths

As a starting point, while Malawi has previously had a very high maternal mortality ratio (MMR), this number has fallen significantly in recent years. While the WHO estimated an MMR of 749 in the year 2000, their latest estimate for 2017 has more than halved to 349, equalling 2100 maternal deaths [3]. The Global Burden of Disease (GBD) study is perhaps the other major source for MMR estimates, described by *The Lancet* as “the most comprehensive worldwide observational epidemiological study to date” [4]. In 2015, the GBD study, published in *The Lancet*, estimated 1462 maternal deaths in Malawi, with an MMR of 219.7 [5]. The latest results from the GBD 2019 study, published on the GBD website, suggest 1150 maternal deaths and an MMR of 211 [6]. While higher MMR figures than this have been cited in the past, the latest evidence from the WHO and GBD suggests between 1150 and 2100 maternal deaths per annum, with the lower figure being a more recent estimate.

While some have suggested using Demographic and Health Surveys (DHS) as the basis for maternal mortality estimates, this is questionable. The DHS Program explicitly advises that in their data, “Maternal mortality rates and ratios are subject to high levels of relative sampling error due to their relatively rare occurrence” [7]. Regarding cause of maternal death, the DHS Program typically does not collect these data at all: “DHS surveys do not include questions that could possibly lead to such estimates” [8]. Hence, DHS data are not included in this review.

## 3. Proportion Attributable to Induced Abortion and Spontaneous Abortion Combined

The most common estimate for mortality from abortive outcomes combined is the claim that 6–18% of maternal deaths in Malawi are attributable to abortion [9], some organisations using the upper bound of this estimate [10], and the figure often being misrepresented as referring only to illegal induced abortions. With 1150–2100 maternal deaths annually, this would amount to 69–378 deaths a year. Since there are no credible estimates above 18% for proportion attributable to abortive outcomes, and no recent estimates above 2100 for total maternal deaths, any estimate exceeding 378 has no empirical basis. To narrow down this estimate to a more precise figure requires an examination of the existing sources on this question.

The Guttmacher Institute claim that 6–18% is a ‘recent’ estimate [9]. It is worth exploring just how recent the figure is—especially the upper bound—since maternal mortality attributable to abortion changes significantly over time as healthcare systems develop [11,12,13], including in Malawi [14,15]. Mortality from induced abortion dropped to minimal levels before abortion was legalised in most Western countries, for example, and is minimal to non-existent in high-income countries where abortion remains mostly illegal, such as Chile, Poland, Malta, (pre-legalisation) South Korea, (pre-legalisation) Ireland and across Northern Africa and Western Asia. The proportion of maternal deaths due to abortive outcomes is approximately the same in developed regions—7.5%—as in developing regions, even though abortion is almost entirely legal in the former and mostly illegal in the latter [11]. As illegal abortion becomes safer [12,16,17], and as post-abortion care—which is usually technically simple—improves [13,18,19,20], the proportion of maternal deaths due to abortion is likely to be significantly lower now than it was 10 or 20 years ago.

The 6–18% figure cited by the Guttmacher Institute and elsewhere comes in turn from Polis et al.’s 2017 paper [21]. This paper, however, did not make any direct estimate of maternal deaths attributable to abortion, instead citing Eveline Geubbels’ 2013 review of multiple earlier studies [14]. This is the primary source of most estimates of abortion mortality in Malawi. Indeed, it is difficult to find any recent sources which do not ultimately rely on Geubbels’ work, so it will be instructive to consider the basis of her estimate.

Geubbels cites 6 studies, 3 of which give a figure of 17–18% of maternal deaths being attributable to abortion [22,23,24]. These three studies, however, all date from 1989 and 1990, over one-quarter of a century prior to the Guttmacher claim, during which time maternal mortality and its causes have changed drastically in Malawi and across the world. Two had samples only from one hospital. There is no more recent study supporting a figure close to 18% of maternal deaths.

Geubbels then discusses more recent studies, but these all offer significantly lower figures, and even these data are now quite old. The next study cited by Geubbels used data from one district from 1998 to 2001, with a sample size of 43, finding that 14% of deaths (6 deaths in total) were due to abortive outcomes [25]. This study, however, appears to be superseded by the 5th study, which had one of the same co-authors and covered a much larger sample size (312) from 18 different hospitals across Southern Malawi from the year 2001. This study found that only 6% of maternal deaths were attributable to abortive outcomes [15]. Finally, the sixth and most recent study cited by Geubbels, the Farish community survey, is from 2003, but is not cited and the present author has been unable to identify it. Regardless, it does not appear to record any deaths from abortion at all in Geubbels’ review, so can be discarded.

In summary, the most recent identifiable study from Geubbels’ review was 16 years old when the Guttmacher claim was originally published, and is now 20 years old. This paper claims only 6% of deaths due to abortion and includes deaths from miscarriage. By contrast, the data suggesting figures closer to 18% are now more than 30 years old and cannot form a reliable basis for contemporary estimates.

There is a distinct paucity of peer-reviewed studies identifying causes of maternal mortality in Malawi since Geubbels’ review. One study looking at 43 deaths across 9 hospitals in 2007 found abortive outcomes to account for only 3 deaths, approximately 7% [26]. Of course, given the small sample size, this will have a large confidence interval when extrapolated to a long-term population estimate. Table 1 summarises these studies.

Hence, the latest evidence, which is now itself 15–20 years old, suggested that by this time, only 6–7% of maternal deaths were due to abortive outcomes. Given contemporary MMR estimates, this would amount to approximately 70–150 deaths a year depending on the MMR estimate. As post-abortion care has improved and safer methods of illegal abortion have become available, this proportion has likely decreased further still. However, this could be offset if abortion numbers have increased; recent, reliable data on which are lacking. More recent data distinguishing the causes of maternal mortality in Malawi are unavailable and would be helpful for taking this debate forward. Figures well below 10%, as suggested by the most recent data, would fit with more recent data from Uganda suggesting 5% [27], and Rwanda suggesting 3% [28] of maternal deaths attributable to abortive outcomes prior to legalisation. In Ethiopia, abortive outcomes had fallen to 6–7% of maternal deaths prior to legalisation in 2005, though have not decreased further, and may have increased, since legalisation [29,30,31,32].

An alternative estimate for maternal mortality attributable to abortion comes from Otland et al. in 2014. Otland et al. claimed that unsafe abortion was responsible for 24–30% of maternal deaths in Malawi [33]. However, they cite only Jackson’s 2011 review [34] and an unpublished oral presentation. Jackson in turn cites the same unpublished oral presentation, Geubbels’ major review and an unpublished meeting held by Ipas. None of these sources are verifiable other than Geubbels’ review, which we have discussed at length and which at no point—not even 30 years ago—described figures such as 24–30%. It remains unclear where the 24–30% figure comes from. There remains, therefore, no basis in the peer-reviewed academic literature for estimates this high.

## 4. Distinguishing Spontaneous from Induced Abortion

A major—perhaps the major—issue with estimating deaths from unsafe abortion is that in mortality statistics, ‘abortion’ almost invariably refers to both induced abortion and spontaneous abortion (and sometimes also to ectopic pregnancy [11]). It is rare that studies attempt—or are even able—to differentiate induced from spontaneous abortions. Especially with the advent of medical abortion, induced and spontaneous abortion complications present similarly, and it is sometimes impossible to tell whether an abortion is induced or spontaneous. In other cases, there may be evidence in one direction or the other: cervical lesions from self-induced abortion, for example, or clear evidence of a known, wanted pregnancy prior to the complications (since most clandestine abortions will be attempted before the pregnancy is widely known). It may not be assumed that all, or even most, of these deaths are from induced abortion.

Among those studies which do attempt to differentiate deaths from spontaneous and induced abortion, many find that the majority of deaths from abortion are in fact spontaneous, and hence have nothing to do with induced abortion [35,36,37,38,39,40,41]. For example, the Royal College of Obstetricians and Gynaecologists estimated that before abortion was legalised in the UK, fewer than 20% of deaths from abortion were from induced abortion—the rest being spontaneous [35]. Methods which aim to estimate proportions on the basis of natural miscarriages typically involve highly questionable assumptions: for example, that miscarriage rates are relatively invariant, and that the number of women presenting to hospital with miscarriages is only 3.41% of the number of live births and invariant between contexts—even though significant risk factors for miscarriage are well known [42], and far higher miscarriage hospitalisation rates have been documented in countries with fewer risk factors [43,44]. So it is likely both that (1) deaths from abortion have continued decreasing in Malawi in the last 15–20 years, and (2) some of these deaths—perhaps most—are actually a result of miscarriage. Hence, the proportion of maternal deaths attributable to induced abortion in Malawi is likely very small. At the very least, there is no firm empirical basis for assuming that the majority of these deaths are from induced abortion.

## 5. Discussion and Conclusions

Of course, the small number of women who do die from illegal abortion in Malawi is still important, as these are women with dignity and worth whose deaths are tragedies. However, their numbers should not be radically inflated.

It is usually held that the legalisation of abortion would help save the lives of these women. The evidence for this is, however, surprisingly sparse, given that maternal mortality and abortion mortality naturally fall over time as a result of safer illegal abortion and better post-abortion care [11,12,13,14,15,16,17,18,19,20]. Hence, maternal mortality and abortion mortality decrease in the same way when abortion is criminalised, as in Poland and Chile.

Conversely, liberalisation of the abortion law in Rwanda saw an increase in the proportion of maternal deaths due to abortion [28]. Although it could be suggested that liberalisation led to decreased stigma and increased reporting, this explanation is not plausible because the data concern cause of death from abortive outcomes as a whole, and the *total* sum of these is unchanged regardless of how many induced abortions are reported as spontaneous abortions.

Likewise, legalisation of abortion in Ethiopia saw a significant increase in the severity of complications from abortion, with no decrease in the number of illegal abortions [45,46]. Even if this were due to provider inexperience, as has been suggested, the same would likely be true in Malawi, and the increased number of severe complications in Ethiopia continued for at least 9 years after legalisation [46].

These patterns are by no means unique, and have been seen in industrialised countries as well [35,47]. Section 3 explained that high-income countries with restrictive abortion laws have minimal abortion mortality; by contrast, low-income countries with liberalised abortion laws still have significant abortion mortality [13,46]. The proportion of maternal deaths attributable to abortive outcomes is similar between developed regions and developing regions, despite the general difference in abortion legislation [11]. Once socioeconomic and infrastructural factors are taken into account, there appears to be little to no relationship between abortion legislation and abortion mortality.

A few explanations can be offered for this surprising lack of association: first, when abortion is legalised, more women obtain abortions [48,49,50], putting more women at risk. In some cases, legalisation even resulted in increased illegal abortions [50,51]. Second, legal abortion appeals to a different clientele than those seeking illegal abortions—even when abortion is legalised, many women continue seeking illegal abortions for various reasons, including privacy, despite access to legal abortion [51,52,53,54,55]. Third, good-quality post-abortion care is usually sufficient to prevent abortion mortality [13,18,19,20]. Finally, legal and illegal abortion are both converging towards self-managed medical abortion [12,16,17,41,56]. Legal medical abortions may carry increased risks of haemorrhage [57] given the limited opportunity to examine the patient for signs of anaemia, ectopic pregnancy and gestation through telemedicine consultations, and the high prevalence of anaemia and severe anaemia in low- and middle-income countries, particularly where malaria is common [58,59].

Since legalising abortion can make it safer in some ways, but can encourage more women to have abortions in the first place, the effect of legalised abortion on abortion mortality can only be determined by rigorous analysis of the empirical evidence. At present, that evidence suggests mixed outcomes. The effect of abortion legalisation on maternal mortality generally is far more complex still, and beyond the scope of this paper.

Understanding the causes and tractability of maternal mortality is critical to reducing it, especially if large reductions are sought. There has been significant criticism of aid programmes focusing too much on family planning and not enough on basic obstetric care in Malawi: the UK government’s aid watchdog found that the UK government disproportionately prioritised family planning at the expense of strengthening health systems, and, as a result, Malawi had made ‘no progress’ in reducing maternal mortality: ‘many women are still dying from basic obstetric complications … progress on improving emergency obstetric and neonatal care has been well short of targets. In Malawi, by 2016 15% of health facilities were able to provide basic emergency care, against a target of 30% … shortfalls in progress on improving the quality of maternal health services meant that reductions in maternal mortality were significantly below what they could have been’ [60]. The watchdog noted also that even the estimates for lives saved through family planning were highly exaggerated. This accords with a *BMJ* cost-effectiveness analysis noting that the data on cost-effectiveness of family planning is too limited to make an assessment [61]. Research evaluating UK aid interventions to reduce maternal mortality in Malawi found significant emphasis on liberalisation of abortion laws in Malawi, with *The Lancet* later publishing the authors’ criticism of the UK Department for International Development and their implementing advocacy partners (Ipas and Marie Stopes International) for censorship, interference and career threats, noting the strong incentives to ‘show that their investments deliver results’, potentially exaggerating programme effectiveness [62,63]. A subsequent paper in *BMJ Global Health* noted that this was a common phenomenon, potentially having an enormous impact on cost-effectiveness and impact evaluation research [64].

The causes of maternal mortality identified in the latest two studies in Malawi are presented in Table 2, along with the latest WHO/Lancet estimate for sub-Saharan Africa more generally.

Since haemorrhage and sepsis are jointly responsible for 31–47% of maternal deaths, these should be the specific conditions to be prioritised. Nevertheless, there are relatively few interventions for specific conditions which have proven cost-effectiveness [65]. In general, think tanks and reviews focused on cost-effectiveness emphasise improving basic infrastructure, emergency obstetric care access, and wide coverage of basic provisions [61,65,66]. This is reinforced by the large and increasing [59] proportion of indirect maternal deaths, which are commonly neglected, but which further emphasise the importance of general healthcare system strengthening, since most in Malawi are due to HIV/AIDS, anaemia, malaria, and, in the past, meningitis (many of which were, in turn, due to HIV/AIDS).

For these reasons, resources aiming to reduce maternal mortality in Malawi should be spent primarily on improving access to basic emergency obstetric care and other infrastructural development: “It may be worthwhile to scale down implementation of less cost effective interventions … and to reallocate these resources to more cost effective options such as community based newborn packages, selected antenatal care, and skilled attendance [at birth]” [61].

In sum, the latest evidence suggests that 6–7% of maternal deaths—therefore approximately 69–147 per annum depending on estimate of total maternal deaths—in Malawi are due to abortion and miscarriage combined. The more recent estimate of maternal mortality suggests the lower end of this range. Even these percentages, however, are now two decades old, and have likely decreased further given significant advances in the safety of illegal abortion and quality of post-abortion care.

A large number of these deaths—perhaps even a majority—are from miscarriage, and hence only a very small percentage, and a very small number, of maternal deaths are due to induced abortion specifically. The evidence that this small number of women would have been saved by legalisation of abortion remains sparse. As a result, to reduce maternal mortality with limited resources, emergency obstetric care and wide coverage of basic healthcare infrastructure should be prioritised over costly, controversial and likely ineffective policy advocacy.

Claims in the mainstream media of thousands of women [67,68] dying from unsafe abortion in Malawi each year have no empirical foundation. This has, unfortunately, not stopped them being repeated by reputable medical bodies, with the Royal College of Obstetricians and Gynaecologists recently endorsing a claim that 12,000 women in Malawi die from unsafe abortion each year [69]. Medical authorities should refrain from making false or outdated claims about abortion mortality in Malawi, and from using this as an argument for legal reform. Instead, it is critically important that abortion debates in Malawi are based on the most recent scientific and medical evidence. This paper has summarised the evidence base from the 1990s to the current day on this question.

## Figures and Tables

**Table 1:** Summary of the existing primary studies on proportion of maternal mortality attributable to abortion. ‘Abortion’ here refers to illegal induced, legal induced, and spontaneous abortions combined. Studies are referenced in the bibliography.

Study	Abortion Mortality (% of Maternal Deaths)	Year of Data Collection	Sample Size
Driessen (1990) [22]	18%	1989	214
Wiebenga (1992) [23]	17%	1989–1990	151
Sangala (1992) [24]	18%	1990	74
Hofman (2005) [25]	14%	1998–2001	43
Ratsma (2005) [15]	6%	2001	312
Farish (2003) [14]	-	2003	-
Kongnyuy (2009) [26]	7%	2007	43

**Table 2:** Primary causes of maternal death in Malawi and Sub-Saharan Africa. Studies are referenced in the bibliography.

Study	Haemorrhage	Sepsis	Ruptured Uterus/Obstructed Labour	Hypertension	Abortive Outcomes	Indirect Causes	Year of Data Collection
Ratsma (2005) [15]	10.9%	20.4%	15.5%	5.3%	6.6%	35.2%	2001
Kongnyuy (2009) [26]	30.2%	16.3%	7.0%	4.7%	7.0%	34.9%	2007
Say (WHO) (2004) [11]	24.5%	10.3%	Not given	16.0%	9.6%	28.6%	2003–2009

## Data Availability

Not applicable.
